# Real-world statistics at two timescales and a mechanism for infant learning of object names

**DOI:** 10.1073/pnas.2123239119

**Published:** 2022-04-28

**Authors:** Elizabeth M. Clerkin, Linda B. Smith

**Affiliations:** ^a^Department of Psychological and Brain Sciences, Indiana University, Bloomington, IN 47405-7007;; ^b^Cognitive Science Program, Indiana University, Bloomington, Bloomington, IN 47405-7007;; ^c^School of Psychology, University of East Anglia, Norwich NR4 7TJ, United Kingdom

**Keywords:** language acquisition, object names, input statistics, infancy

## Abstract

Infants begin learning the visual referents of nouns before their first birthday. Despite considerable empirical and theoretical effort, little is known about the statistics of the experiences that enable infants to break into object–name learning. We used wearable sensors to collect infant experiences of visual objects and their heard names for 40 early-learned categories. The analyzed data were from one context that occurs multiple times a day and includes objects with early-learned names: mealtime. The statistics reveal two distinct timescales of experience. At the timescale of many mealtime episodes (*n* = 87), the visual categories were pervasively present, but naming of the objects in each of those categories was very rare. At the timescale of single mealtime episodes, names and referents did cooccur, but each name–referent pair appeared in very few of the mealtime episodes. The statistics are consistent with incremental learning of visual categories across many episodes and the rapid learning of name–object mappings within individual episodes. The two timescales are also consistent with a known cortical learning mechanism for one-episode learning of associations: new information, the heard name, is incorporated into well-established memories, the seen object category, when the new information cooccurs with the reactivation of that slowly established memory.

## Significance

Infants learn mappings between heard names and seen things before their first birthday and before they produce spoken language. Two challenges to explaining this early learning are the immaturity of infant memory systems and the infrequency of any individual object name in the heard language input. We quantified the frequency of visual referents, heard names, and the cooccurrences of referents and names in infant everyday experiences. We discovered statistical patterns at two timescales that align with a cortical mechanism of associative memory formation that supports the rapid formation of durable associative memories from very few experienced cooccurrences.

Traditional theories of language learning assumed a “poverty of the stimulus”: the input is noisy, imperfect, and insufficient for learning without domain-specific constraints and powerful inferential mechanisms (1–3). Despite many empirical studies, elegant models, and specific advances, the poverty of the stimulus is still a major theoretical barrier, and the field has no clear understanding of how infants break into language or solve even the earliest components of language learning, including how they learn their first object names. Here we provide evidence on the statistics of infants’ everyday encounters with object names and their referents. The findings confirm the limited nature of the input but also reveal statistical properties that align with a known learning mechanism, albeit one that has not been explicitly considered as an explanation of infant word learning.

Infants begin learning object names well before their first birthday and before they produce spoken language. This learning is evident in laboratory findings in which infants as young as 6 mo of age preferentially look to pictures of an object upon hearing the object’s name (4–7; but see ref. 8). However, there is little evidence documenting the everyday experiences through which infants could form these initial object–name mappings. This evidence has been difficult for researchers to obtain because day-in and day-out language experience is massive, but individual words occur sparsely in any given sample of that massive input (9). By some estimates, the average child hears 20,000 word tokens a day or over 7 million word tokens in a year (10, 11), which may seem like rich rather than poor input. However, the frequency distribution of individual words in any sample is characterized by a power law (9, 12, 13); a very few words (function words, some light verbs) are very frequent, but most content words are individually rare. Object names, even common and early-learned ones, fall in the long tail of the frequency distribution of words in large corpora of parent speech to children that aggregate parents’ speech across many different contexts (9, 13–15). The absolute frequency of early-learned object names even in these large corpora of child-directed speech is quite low. Moreover, the temporal distribution of individual words is neither evenly nor randomly distributed in time. Rather, it is bursty and tied to context (9, 16–18). For example, speech about bowls is more likely at breakfast than when playing in the park. Thus, the measured frequency of any individual word in a sample of infant-directed speech can vary markedly from context to context and differ considerably from the base rate for that word when calculated from large collections of speech or text (9, 19, 20).

Amid these difficulties, researchers have attempted to characterize the learning environment by sampling speech within the home (for an hour up to whole days) and measuring the object names heard, their contexts, and the probability that the referent was present given the spoken name (e.g., refs. 4 and 21). Because of the sparsity of any specific object name in a sample of language, researchers typically report aggregate statistics computed across all object names in the sample: that is, reporting the rate of heard name–referent cooccurrences combined over all the different object names uttered in the vicinity of the child during the sampled input (4, 21–23). These studies consistently show that the aggregate cooccurrence statistics of visual referents and their names when computed across many different words predict individual infants’ current (4) and later vocabulary sizes (23). These studies do not, however, specify the available experiences for learning any individual object name. The specific goal of the present study was to quantify the statistics of individual object names and referents for a set of early-learned names in a corpus representing the everyday environments of infants. Given the context-dependent distributional properties of object names in speech, we focused on one context, mealtime, that occurs multiple times a day for infants and that commonly involves objects with names that are among the earliest learned in American English (24).

Our approach to quantifying the name–object statistics in the infant learning environment was motivated by a specific candidate learning mechanism. In experimentally controlled studies, infants under a year of age show their early learning of name–referent mappings by looking to a pictured referent over a foil upon hearing the name (4–7). To do this, infants must have formed and retained an association between the name and the visual properties of the referent. According to the Complementary Learning Systems model of memory (25, 26), there are two paths through which durable and expressible associative memories may be formed. The first path operates with hippocampal involvement and can lead to lasting learning from a single episode of experience. Recent evidence (27) indicates that the hippocampus is active during learning tasks in infancy, but the extant evidence also suggests immaturities that may make this path insufficient for forming durable memories from a single experience in infant brains (28–30). The second path operates within the neocortex but was originally thought to form durable memories only gradually, after many repeated experiences (26). However, contemporary research shows that this path can lead to one-episode memory formation under special conditions: A new association can be rapidly formed from one or a few experiences of the cooccurring components if there is an already well-established memory of one component and if this well-established memory is reactivated in the context of the new information (i.e., refs. 31–36).

**Fig. 1.**
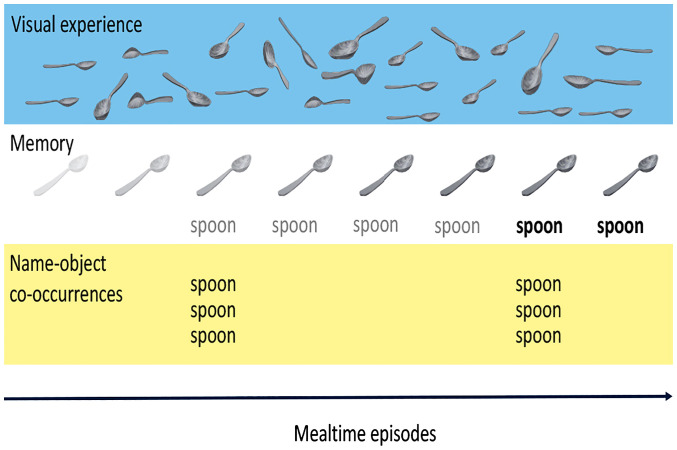
An illustration of the hypothesized two-timescale statistical pattern and its potential role in rapid associative memory formation. Pervasive visual experiences build robust memory for visual object categories. Rarer episodes, including both the name and a referent, are integrated into the established memory.

This form of rapid memory formation is a plausible candidate mechanism for early object–name learning that could work despite the sparsity of any specific to-be-learned object names. This path (Fig. 1) to learning would require the potential referents of early-learned nouns (i.e., objects) to be pervasively present in everyday experiences (37) so that durable visual memories of the to-be-referents could be incrementally formed. This path would also require the reactivation of established object memories on the rarer occasions when the name is heard so that the new information (i.e., the name) could be integrated into the established memory of the object category. With this candidate mechanism in mind, we analyzed a corpus of egocentric audiovisual recordings collected by sensors worn by infants during mealtimes. We estimated the frequency of heard names for a set of normatively early-learned object categories, the frequency of visual referents in the infants’ field of view (FOV), and the frequency of name–object cooccurrences at two timescales. We first measured these properties across all the mealtimes episodes, providing an estimate of the quality of the input with respect to the incremental formation of visual referent memories from repeated mealtime experiences. We then measured these properties within individual mealtimes, providing evidence on the expected rarer name–object cooccurrences within an episode that could lead to the rapid formation of a name–referent memory given past pervasive visual experience with the referent category.

## Results

The mealtime corpus consists of 87 mealtime events (mean duration = 11.22 min, SD = 11.87), which were contributed by 14 infants (mean age = 9 mo, SD = 1.33) who wore head cameras with audio recording capability at home for multiple portions of the day across several days. The infants collected a combined total of 67.1 h of home video from which the mealtime segments were extracted (see *SI Appendix*, Table S1 for recording information per participant). Mealtimes were defined as any time when food or eating (by anyone) was present in the head-camera video. An interest in mealtimes was not expressed to the parents at the time of recording, nor was an interest in object names.

### Forty Categories.

**Fig. 2.**
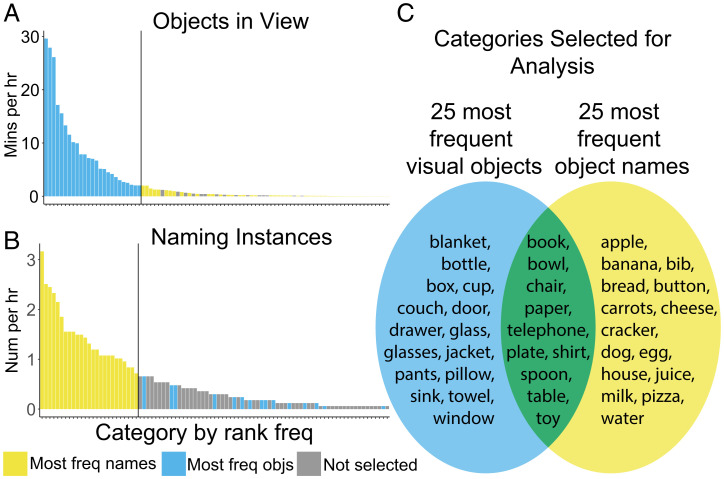
Corpus frequency as referents and names and the selection of categories for further analysis. (*A*) The visual frequency in minutes in view per hour for each of the initial 89 categories rank-ordered by frequency. Vertical line indicates the cutoff point between the 25 most visually frequent objects and those not chosen for further analysis. (*B*) The lexical frequency in naming instances per hour for each of the initial 89 categories, rank-ordered by frequency. Vertical line indicates the cutoff point between the 25 most lexically frequent objects and those not chosen for further analysis. A list of the 89 categories and the frequency of their names and referents is available in *SI Appendix*, Table S2. (*C*) A Venn diagram showing the 40 categories selected for further analysis and whether they were one of the 25 most visually frequent, 25 most lexically frequent, or both. Visual frequency was determined from 11,549 images and yielded 49,578 instances of the 89 objects. Naming frequency was determined from 12,050 analyzed 5-s speech segments and yielded a total of 873 naming instances.

Through a two-step process, we identified object categories with sufficient visual and heard-word experiences to be potentially learnable at mealtimes. First, we created a larger candidate set of categories that had normative receptive ages of acquisition before 18 mo (24, 38) and that were visually present at least once and occurred as a heard name at least once in the mealtime corpus. There was no requirement that the referent and name occur in the same mealtime. There were 89 categories that met these criteria (*SI Appendix*, Table S2). Second, from this candidate set, we selected the 25 categories for which the visual referents were most frequent (total minutes in view in the corpus) (Fig. 2*A*) and the 25 categories for which the heard names were most frequent (total name occurrences in the corpus) (Fig. 2*B*). These two lists were generated independently of each other, and 10 categories appeared on both lists because they met the criterion both for visual and for speech frequency (Fig. 2*C*). This selection approach thus yielded the 40 total categories (Fig. 2*C*) with the most frequent occurrences for each side of the learning problem: the seen thing and the heard name. Measures were computed at the category level (aggregating across subjects). Statistical analyses, unless otherwise noted, were computed with the categories (and not subjects) as the unit of analysis.

The frequencies of the visual referents and the frequencies of their names for the 40 categories (Fig. 2) were not significantly correlated (*rs* = −0.25, df = 38, *P* = 0.14).* Consistent with the scale-invariant distributions of objects in environments (37) and words in spoken language (12, 39), the frequency distributions of the visual referents and their names (Fig. 2 *A* and *B*) were nonnormally distributed with skewness of 1.76 and 0.74, respectively (SEs = 0.37). Thus, within the corpus, some objects and some names were frequent and others were infrequent, making central tendency measures across the 40 categories potentially misleading. Accordingly, we present evidence on object frequency, name frequency, and cooccurrence for all 40 categories and also present the same statistics separately for subgroupings of the 10 most visually frequent and the 10 most frequently named categories.^†^

By definition, the selected categories have early-learned names: that is, names that are normatively in the receptive vocabularies of 50% of infants at or before 18 mo of age (range: 8 to 18 mo), as reported in the Wordbank database (38). A Kruskal–Wallis *H* test did not find a significant difference between the age of receptive acquisition of the names for visually frequent, lexically frequent, nor both visually and lexically frequent categories [*H*(2) = 1.87, *P* = 0.39]. The visually frequent categories included background fixtures (e.g., windows, tables, doors), small manipulable object (e.g., plates, cups, spoons), and other items generally common in the lives of infants. Many of the frequently named items are foods. The 10 categories that were on both the visually frequent list and the lexically frequent list were a mix of kinds of categories.

### Statistics for Incremental Learning.

Incremental memory formation is believed to require repeated experiences distributed across hours and days (26). To quantify the input relevant to this learning path, we considered mealtimes as discrete events and measured the frequency of the relevant components for learning—referent, name, and name-plus-referent—as the proportion of mealtimes in the corpus in which the measured component occurred at least once. We used this low threshold (occurring once in a mealtime) for two reasons. First, the goal was to estimate the repetition of components over discrete time-separated events. Second, the proportion of mealtimes in which a component occurred at least once is a comparable metric for visual and lexical presence, which are otherwise difficult to compare given the vastly different timescales at which they occur. Visual objects can be present in an infant’s FOV for a range of durations (from a few seconds to fractions of hours), whereas an object name spoken aloud is always very brief (a fraction of a second).

**Fig. 3.**
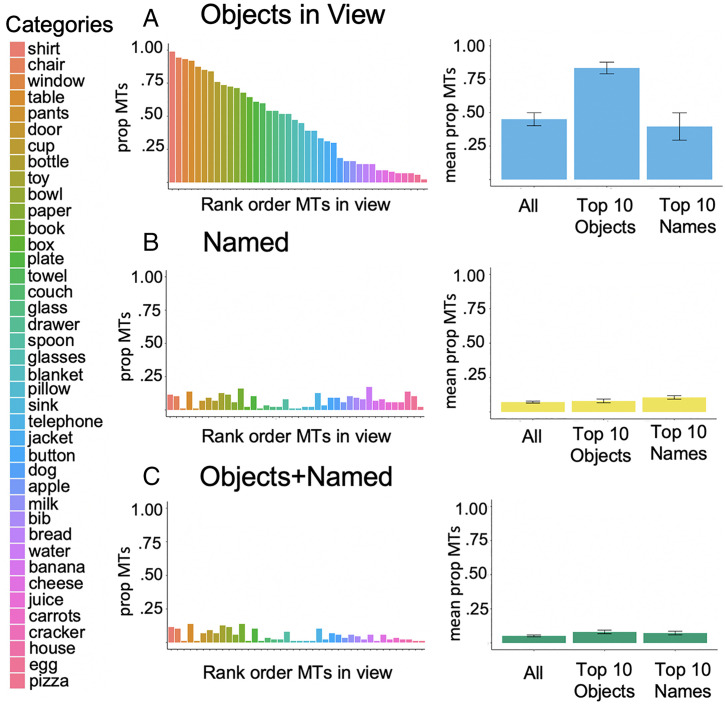
Frequency of the 40 categories as spoken names and visual objects across mealtime events. (*A*) The proportion of mealtime videos for each category in which the visual object occurred in at least one frame and the mean proportion of mealtimes in which the visual object occurred for all 40 categories, the top 10 objects, and the top 10 names. (*B*) The proportion of mealtime videos for each category in which the object name was said at least once and the mean proportion of mealtimes in which the object name occurred for all 40 categories, the top 10 objects, and the top 10 names. (*C*) The proportion of mealtime videos for each category in which the visual object and the object name both occurred at least once and the mean proportion of mealtimes in which both the visual object and the object name occurred for all 40 categories, the top 10 objects, and the top 10 names. Error bars represent SEM. For the 40 categories, there were 46,309 instances of the objects in view and 678 naming instances.

By this count-of-episodes metric (Fig. 3 *A* and *B*), visual referents for the 40 categories were present in substantially more individual mealtimes than were the spoken names (Wilcoxon signed-ranks test, *z* = −5.09, *P* < 0.0001). The mean proportion of mealtimes in which referents were present for the 40 categories was 0.45 (SD = 0.31, median [Mdn] = 0.46, interquartile range [IQR] = 0.58), whereas the mean across the corresponding category names was 0.07 (SD = 0.04, Mdn = 0.07, IQR = 0.08). There is thus a greater than 6:1 ratio of mealtimes with the referent present to mealtimes with the name present. This imbalance in frequency also holds as significant for the top 10 categories in visual frequency (*z* = −2.75, *P* < 0.05); for this group, the mean proportion of mealtimes with the visual referent present was 0.83 (SD = 0.14, Mdn = 0.86, IQR = 0.15), and the mean proportion of mealtimes with the name present was 0.08 (SD = 0.04, Mdn = 0.09, IQR = 0.04). The difference was marginally significant for the top 10 categories in name frequency (*z* = −2.04, *P* = 0.07); for this group, the mean visual presence was 0.4 (SD = 0.32, Mdn = 0.34, IQR = 0.56) and the mean proportion of mealtimes with the name present was 0.11 (SD = 0.04, Mdn = 0.1, IQR = 0.05), In brief, the structure of the mealtime environment is one in which the visual referents for early-learned categories are persistently present, but the heard names are not.

Because names occurred in very few mealtimes, cooccurrences of individual names and a visual referent also occurred only in a very few mealtimes for each category (Fig. 3*C*); the proportion of mealtimes in which both the name and the corresponding visual referent occurred was low across categories (mean = 0.05, SD = 0.04, Mdn = 0.03, IQR = 0.06) and for the individual categories. If incremental learning of visual object categories requires repeated visual experiences over multiple time-separated episodes, then there appear to be sufficient experiences for learning the visual referent categories. In contrast, if infant learning of object names requires repetitions of name–referent cooccurrences distributed over multiple time-separated events, then even the most frequent object names present in this mealtime corpus would appear difficult to learn. Yet all the object names in the analyzed corpus are normatively early-learned and refer to objects common at mealtimes. Current understanding (40) suggests that a durable memory of a name–object association could be formed within a single mealtime if a well-established memory of the visual referent was reactivated in the context of the heard name.

### Statistics for Rapid Name–Referent Learning.

If infants have a robust representation of the visual referent built up across time-separated mealtime events, then reactivation of the memory close in time to when an object name is heard may be enough to form a durable memory that binds the name to the properties of the referent (40). If these statistics hold, then early object names may be readily learned by young infants despite the rarity of the individual object names. Accordingly, this set of analyses focused on mealtimes in which the name of one of the 40 categories was present and in which the visual referent was also present. Although the overall proportion of mealtimes in which a referent and its name cooccurred was low, the average proportion of the mealtimes in which the name was uttered that the referent was also present was high at 0.74 (SD = 0.29, Mdn = 0.84, IQR = 0.5). Within the mealtime corpus, common objects were persistently present, whether names were present or not, and thus when the names did occur, a referent object was typically also present.

**Fig. 4.**
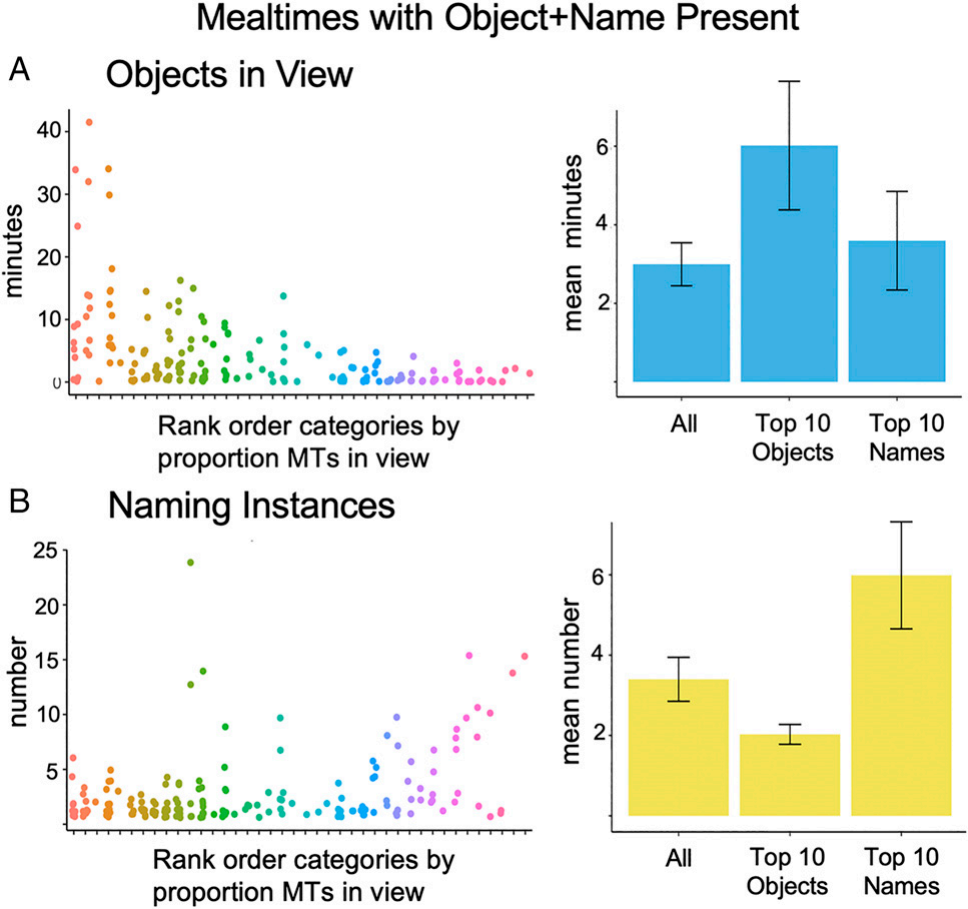
Frequencies of objects and names when both were present within the same mealtime. (*A*) The minutes in which the visual object was in the infants’ field of view in each mealtime in which the name also occurred for each of the 40 categories and mean minutes the visual object was in view per hour for all 40 categories, the top 10 visual objects, and the top 10 object names. (*B*) The number of naming instances in each mealtime in which the referent also occurred for each of the 40 categories and the mean naming instances all 40 categories, the top 10 visual objects, and the top 10 object names. Error bars represent SEM. These data were determined from the 9,485 instances of the objects in view and 504 naming events that occurred within mealtimes in which both the visual referent and the name occurred.

When both the referent and name were present in a mealtime, the referent was in the infant’s FOV (Fig. 4*A*) for 15.6% of the head camera images, or on average for 2.99 min (SD = 3.46 min, Mdn = 1.63, IQR = 3.78). For the top 10 most visually frequent categories, the mean duration of the visual presence of the referent in the infant’s FOV in mealtimes in which the referent was named was 6.02 min (SD = 5.18, Mdn = 4.83, IQR = 6.35) and for the top 10 most frequently named categories, the mean duration of visual presence in mealtimes in which the referent was named was 3.59 min (SD = 3.97, Mdn = 2.56, IQR = 3.83). Experimental studies with infants indicate that these durations of the visual referents should be sufficient to reactivate well-established visual memories (e.g., ref. 41).

For the 40 categories, object names (Fig. 4*B*) were repeated on average 3.4 times within single mealtimes during which their referent was present (SD = 3.46, Mdn = 2, IQR = 2.49); however, there was considerable within- and across-category variability. When named and visually present, the top 10 most visually frequent categories were named an average of 2.03 times within a single mealtime (SD = 0.78, Mdn = 1.9, IQR = 0.7); the top 10 most lexically frequent categories were named on average 5.99 times within mealtimes during which their referent was present (SD = 4.21, Mdn = 3.9, IQR = 4.48). Critically, for 23 of the 40 categories, there was at least one mealtime episode in which the referent was present, and the name was said at least four times, and for 33 of the 40 categories there was at least one mealtime in which the referent was present and named twice. If pervasive experiences with the visual referents form memories that when reactivated allow infants to integrate new information into those durable memories, then the statistical and temporal structure of seen objects and heard names across and within individual infant mealtimes may signal the learnability of name–category associations from few name–object cooccurrences.

### Individual Categories.

**Fig. 5.**
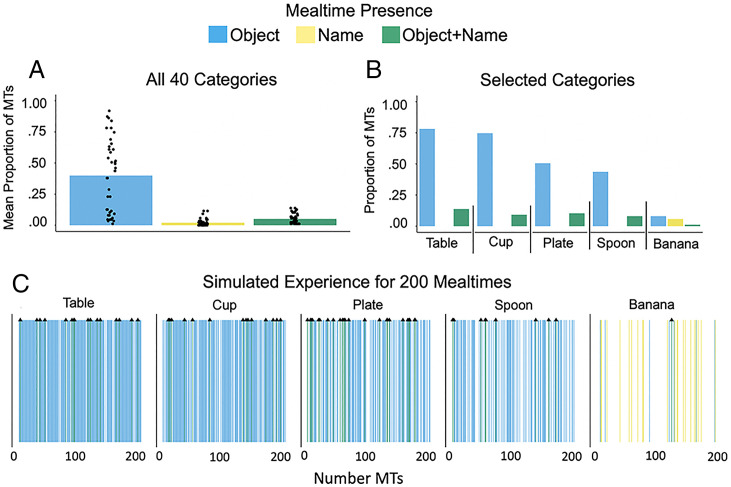
Measured and simulated experience. (*A*) The average proportion of mealtimes in which only the visual object, only the object name, and both the visual object and the object name occurred. Dots represent individual categories. (*B*) The proportion of mealtimes in which the visual object alone, the object name alone, and both the object and its name occurred for the categories “table,” “cup,” “plate,” “spoon,” and “banana.” (*C*) Simulation of experience with the five example categories that a child would be expected to have across 200 mealtimes based on random sampling with replacement from our corpus of 87 mealtimes. Black triangles highlight mealtimes in which both the referent and the name occurred in the simulated series of 200 mealtimes. The simulations were based on observed data in *B*. Across the five example categories in the observed data, there were 11,172 instances of presence of the object and 106 naming events.

Overall, the 40 categories show similar patterns (Fig. 5*A* and *SI Appendix*, Fig. S1): visual referents of common early-learned categories are in the infant’s FOV across many individual mealtimes, whereas the names for those categories occur only in a few mealtimes. However, there are also exceptions in the observed data. The categories of “table,” “cup,” “plate,” and “spoon” (Fig. 5*B*) illustrate the modal pattern evident across the 40 categories: repeated mealtimes without naming and only a few mealtimes in which both the heard name and a visual referent were present. The data for the category “banana” (Fig. 5*B*) showed a different pattern: both visual instances of the category and the heard name were rare, and their joint occurrence within a mealtime was even rarer.

To highlight the different statistics for these five categories, we simulated the expected patterns of occurrence across 200 mealtimes for visual referents, heard names, and cooccurrences using the observed proportions of events in which they occurred in the mealtime corpus (Fig. 5*C*). This simulation represents roughly 40 d in the life of an infant. The categories “table,” “cup,” “plate,” and “spoon” present repeated opportunities for visual learning about referents intermixed with rarer mealtimes that also include the object name. For the category “banana,” however, there are many mealtimes at which neither the referent nor the name is present, and compared to the other example categories, there is a larger proportion of mealtimes in which the name occurs, but a visual referent does not appear in the infant FOV. The absolute infrequency of both the visual referent and the heard name would seem to suggest limited opportunity for learning.

However, laboratory experiments show that infants younger than 1 y of age, the same age as those contributing to this corpus, look to pictures of bananas upon hearing the name (5). Moreover, bananas are among the earliest (and favorite) solid foods given to infants in the United States. There are three possible explanations. 1) The repeated referent information needed to form a well-established memory of a banana is much less than indicated for the categories of “table,” “cup,” “plate,” or “spoon.” 2) Learning the name–referent correspondence for banana occurs outside of mealtimes. This second alternative may be plausible in that laboratory tests that show early receptive knowledge of the word “banana” used pictures of whole and partially whole peeled bananas. The images of bananas in the infants' views at the observed mealtimes, in contrast, overwhelmingly contain mashed or cut up pieces of banana rather than whole bananas. 3) Visual referents are not the sole possible memory anchor for rapid learning of object names. This third possibility is the one we believe deserves attention.

Animal research on rapid cortical memory formation indicates that multimodal memories—especially ones involving action and emotion—are particularly powerful for the rapid integration of novel information into an established memory (42–45). Learning the visual properties of whole bananas as well as learning the name “banana” could both emerge from rapid integration of a seen whole banana and the heard name into a well-established memory of the taste, smell, feel, and enjoyment associated with mashed and cut-up bananas. In sum, rapid learning of name–referent mappings through visual memories built up over time-separated events may be important, but the anchor memories for the rapid integration of heard names may be fundamentally multimodal.

## Discussion

Learning in any domain depends on the internal learning mechanisms and on the statistics of the experiences on which those mechanisms operate. For species-important achievements and for rapid learning more generally, it is to be expected that the learning mechanisms and the real-world statistics align. Here we quantified the statistics for heard names and seen referents for 40 early-learned words at two timescales: across the many time-separated repetitions of mealtimes and within the individual time-continuous events. The main findings are these: Visual referents were persistently present at both timescales. The heard names for those referents were sparse at both timescales. However, in the events in which an object name was heard, the referent was very likely also present during that mealtime. These statistics align with a learning mechanism that operates on two timescales: incrementally forming robust memories over multiple time-separated experiences and rapidly integrating new information into the incrementally established memory when that memory is reactivated in the context of new information (33).

The observed statistics pose the need for a new conceptualization of infants’ learning task. Although many current explanations appear plausible with respect to the more advanced learning of older children, they appear unable to fully explain infants’ initial steps in object–name learning. The two dominant theories, hypothesis testing (46, 47) and cross-situational learning (48–50), are challenged by the sparsity of individual object names in the input and by infant immature memory systems (32). The observed statistics indicate plausible paths to extend both classes of theories to align with the statistics of the input. To do so, theories will need to consider not just name–object cooccurrences as the discrete events on which learning is based, but also how separate incremental learning about multimodal referents may be a critical ingredient for rapid learning of a name–referent pairing from just a few discrete cooccurrences. There are clear pathways to revising current theory in this way: hypothesis testing models (51) could represent incremental learning about referents—independent of heard names—as priors, and cross-situational statistical learning models (52) could operate over the strength of reactivated referent memories rather than the referents perceptually available when an object name is heard.

The proposed two-timescale account of how first object names are acquired requires that visual memories of object categories are acquired without labeling. Considerable research shows that infants between the ages of 3 and 13 mo are robust learners of visual object categories from mere exposure without labeling (53–55). Studies of infants as young as 4 to 6 mo also show that infants daily-life experiences with individual objects (e.g., with the family dog) lead to durable visual memories that are reactivated by novel visual instances (e.g., other dogs, other animals) as indicated by the better categorization and discrimination of those novel test items by infants with a family dog than those without (56–59; see also refs. 4 and 5). These findings indicate that infants acquire sufficiently strong and expressible memories of visual categories that those memories could serve the hypothesized critical base for the rapid integration of a heard name into that memory. Other studies with older infants, beyond the first birthday, show that prior visual familiarization with unnamed referents leads to more rapidly formed and longer-lasting memories for subsequently taught name–referent associations (60–62).

If the present reasoning is correct, the consistency of early perceptual experiences of common categories may be a contributing factor to individual differences in vocabulary development, which in turn predicts many later cognitive achievements (63–65). Researchers working to quantify the quality of language-learning environments and to enhance those environments may benefit from moving beyond measuring the number of words and their diversity in the input to considering the consistency and repetition of common contexts and the referents in those contexts (17, 18). There is large literature showing the benefits of consistent household activities (66–68) in infants and children’s social, cognitive, and language development. By hypothesis, the consistency of nonlinguistic regularities across time-separated but oft-repeated routines plays a central role in the initial learning of object names.

Memory formation and integration is an essential component of all aspects of language learning, and thus the two-timescale learning process implicated by the observed word–referent statistics for infants before their first birthday, may also be relevant for understanding later word learning. However, there are good reasons to suspect the statistics observed in the present study and the learning mechanisms they implicate may be more relevant to infants first learning about object names than to later lexical learning. Memory formation, including rapid hippocampal learning, changes considerably from birth to 2 y of age (32, 69). The input statistics change: Parent talk changes markedly with the advancing language production of the infant (70, 71) and with the infant’s increasing autonomy (72, 73). Infants also play a stronger role in selecting and generating the visual statistics of potential referents via their increasing motor skills (74, 75). Development is a process of continuing change, with each advance building on (or being limited by) past experience.

## Conclusion

If theorists focus on heard words as the key input for learning object names, there is a clear poverty of the stimulus. However, the sparsity and context dependency of individual content words is a pervasive fact of human language (e.g., refs. 9, 19, and 76). Thus, the evolutionary-selected mechanism must work well enough given these statistics. The statistics observed in the present study point to a known and conserved mammalian learning mechanism (33), not specific to learning language and likely insufficient to all aspects of language learning, such as syntax and abstract meanings, but that is perhaps sufficient and essential to getting word learning started. The critical next steps are computational models that can make detailed predictions of learning from the proposed two-timescale mechanism and experimental tests of those specific predictions.

## Methods

### Participants.

The goal was to collect a large sample of infant mealtimes for analysis (77). Given the sparsity of names and objects in samples collected from infants during individual contexts, we formed the mealtime dataset by sampling from a relatively small number of infants repeatedly. That is, we gathered data across multiple mealtime events for each of the 14 infants (8 female, 6 male) in our sample.

We were primarily interested in the everyday learning environments of infants who were not yet talking and were at the stage of acquiring their very first object names (as indicated by looking-time experiments: refs. 4 and 5; and so forth). Therefore, we included data collected from infants aged 7 to 11 mo in our sample (mean = 9 mo, SD = 1.33 mo).

The procedures for recruiting, consenting, and data collection were reviewed and approved by the Institutional Review Board at Indiana University, Protocol no. 1505862312.

### Wearable Sensors.

We sought to capture the infant learning environment from the first-person perspective because the only relevant experiences in the environment are those that make contact with the infant’s sensory systems. We therefore used lightweight cameras (Looxcie 2) mounted in hats and centered between the infant’s eyes to collect these data. The camera weighed 22 g, had a diagonal FOV of 75°, had a vertical FOV of 41°, and had a horizontal FOV of 69° with a 200 to infinity depth of focus. Head-mounted eye-tracking data were not collected because it would be unsafe to do so for infants in their everyday home environment with no experimenters present. Research indicates that freely moving infants (as well as children and adults) predominantly look to the midline of the head-centered FOV with eyes and head aligned (78).

### Instruction to Parents.

Parents brought their infants to the laboratory where the parents provided consent for the study and were instructed on how to use the recording equipment. The infant’s hat size was also determined. Researchers then delivered two hat–camera systems to the family’s home, and the parents were asked to record ∼6 h of video and to collect the footage within 2 wk. The average number of recording days per subject was 10.21 (SD = 4.89). The only stipulation for recording was that the child be awake; parents were otherwise free to record in any context or location and at any time of day that suited them. Parents were not told that word learning was of interest, and mealtimes were never specifically mentioned so as to not alter behavior.

### A Corpus of Mealtimes.

To construct the mealtime corpus, all recorded clips featuring eating by infants or others as well as food preparation and cleanup were included from the recordings from the 14 infants. The final sample included only video segments in which both video and audio were available, and all video segments containing mealtime activities that took place during the same recording session were combined into a single mealtime event. The final corpus contained 87 mealtime events with 6.21 mealtime events per subject on average (SD = 1.93). On average, the mealtime events were 11.22-min long (SD = 11.87).

### Data Analysis.

Still images were sampled from the recordings at 0.2 Hz (one image every 5 s), which resulted in 11,549 images for coding. Naïve adult coders labeled the five most obvious objects in each image using basic level nouns (see ref. 37). Four coders labeled each image; therefore, up to 20 objects could have been labeled in each frame. The object labels recorded by the coders were minimally cleaned in the following ways: adjectives were removed (e.g., “baby spoon” was reduced to “spoon”) unless it was part of a compound noun listed in the dictionary (e.g., “highchair”), in which case it remained as a unique object; different forms of the same object label were collapsed (e.g., “cup” and “cups” were both counted as instances of “cup”); finally, words that did not clearly refer to a concrete object (e.g., “color”) were removed and not considered for analysis.

Speech was sampled continuously but was then divided into 5-s intervals for coding of which there were 12,050. The intervals started between 0 and 5 s before the mealtime and ended between 0 and 5 s after it; therefore, we captured some additional video not coded for visual information (∼27 min) and any speech it contained. Trained coders transcribed all speech in the target infant’s immediate vicinity, including speech to the infant, speech to siblings or other children, and speech to adults, but not background speech, such as television. Each transcription was checked manually for errors by a second coder and was subsequently cleaned as described above for the visual data. A naming moment was defined as any time an object name was said, and all naming moments were extracted from the speech transcripts for the names of all the objects that coders identified as present in the visual scenes.

For the event-level measure, an object was considered present in a mealtime if it occurred in at least one frame, and an object name was considered present in a mealtime if it was said at least once. An object–name pair was considered to have cooccurred in a mealtime if the visual object occurred in at least one frame of the mealtime’s images and the object name was said at least once at any point during the mealtime. That is, the object and the name did not need to appear in the same moment. This is a liberal measure of cooccurrence, which we believe is justified given that an infant might hold an image of an object in mind even if it is not in view at the precise moment the name is said and vice versa.

## Supplementary Material

Supplementary File

## Data Availability

Raw video data are not publicly available because it contains information that could compromise the privacy of research participants (e.g., young children and their families). The coded data analyzed for this paper are available at Open Science Framework (OSF), https://osf.io/bk6xp/ (79). Questions may be directed to the corresponding author or to E.M.C.

## Footnotes

* All *P* values have been adjusted for multiple comparisons using the Benjamini–Hochberg correction.

^†^Two categories were among both the top 10 most frequent objects and the top 10 most frequent names. This overlap is due to selecting for visual and name frequency independently.
