# Supplementary material for: Real-world statistics at two timescales and a mechanism for infant learning of object names
Source: Proc Natl Acad Sci U S A. 2022 Apr 28;119(18):e2123239119. doi: 10.1073/pnas.2123239119 (PMC9170168; doi:10.1073/pnas.2123239119)
Supplement: Supplementary File [file pnas.2123239119.sapp.pdf]

PNAS
www.pnas.org

Supplementary Information for

Real-world statistics at two timescales and a mechanism for infant learning of object names

Elizabeth M. Clerkin[a] and Linda B. Smith[a,b,c]

[a] Department of Psychological and Brain Sciences, Indiana University, 1101 E. 10th St., Bloomington, IN 47405-7007

[b] Cognitive Science Program, Indiana University, Bloomington, 1101 E. 10th St., Bloomington, IN 47405-7007

[c] School of Psychology, University of East Anglia, Norwich, Norfolk, UK

Correspondence author: Linda B. Smith

Email: smith4@indiana.edu.

This PDF file includes:

Figure S1
Tables S1 to S2

Fig. S1.

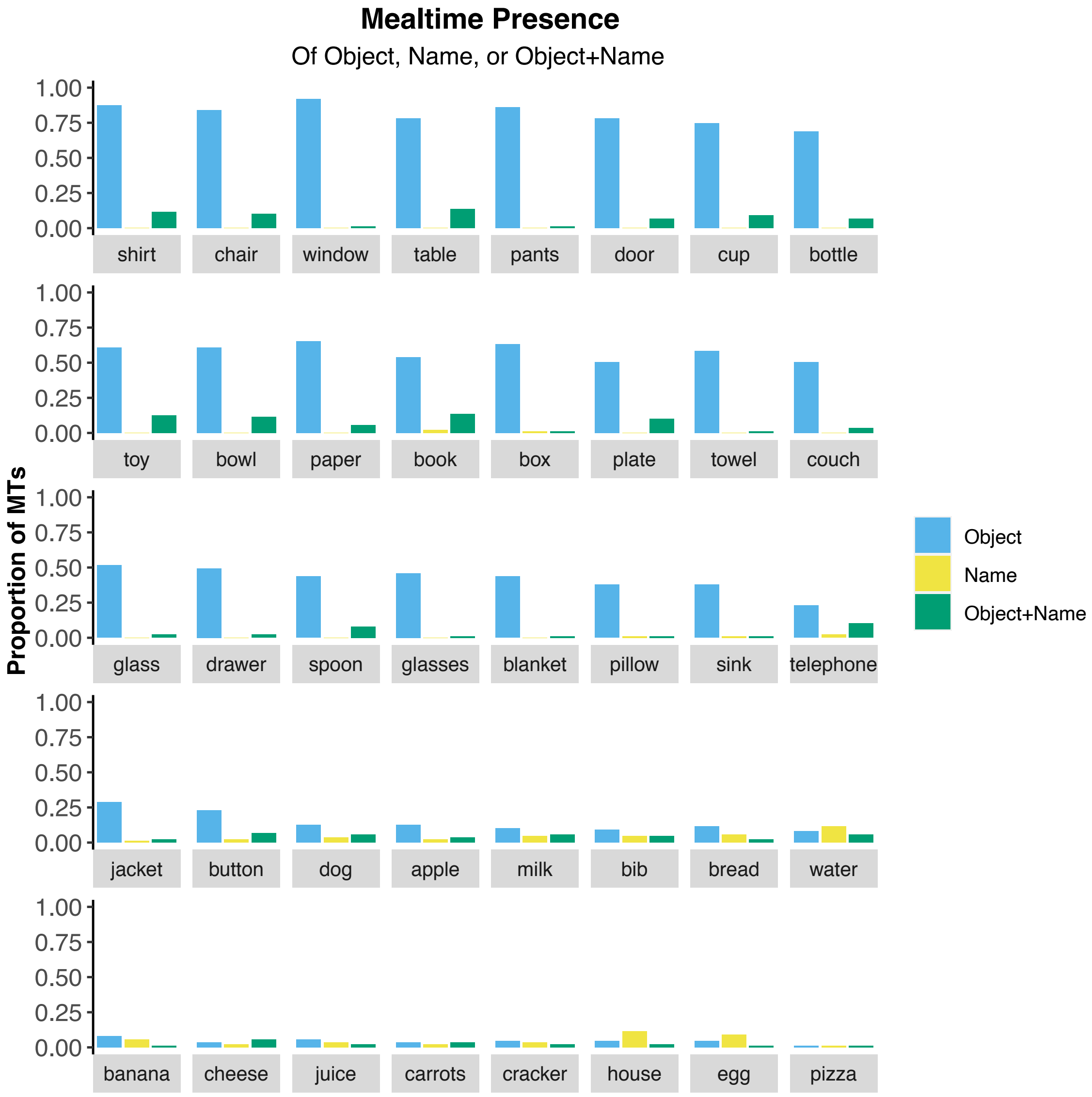

Figure S1. The proportion of mealtimes in which three key events occurred—the referent alone, the name alone, and the referent and the name together for each of the 40 early-learned categories analyzed in the present study.

Table S1. Subject characteristics including age and sex as well as the amount of data contributed by each subject and the number of days taken to record the video footage.

Subject Information					
	Age in Months	Sex	Minutes of Mealtime	Number of Mealtimes	Recording Days
1	7.0	M	184.84	8	9
2	7.2	F	63.54	8	19
3	7.5	F	94.33	6	11
4	7.7	M	41.68	8	16
5	8.4	F	51.96	5	10
6	8.6	F	91.06	6	10
7	8.9	M	74.23	4	2
8	9.1	F	65.47	10	2
9	9.6	F	19.16	4	10
10	9.8	F	90.87	7	9
11	10.0	M	21.45	5	15
12	10.5	M	98.16	7	13
13	10.7	M	33.03	6	12
14	11.0	F	46.78	3	5

Table S2. Early learned categories (n = 89) with at least one naming event and one frame with the visual object present in the corpus. Selection for analysis, visual frequencies (minutes in view) and naming frequencies (naming instances per hour) are provided.

Early-Learned Category Frequency

Category	Selection for Analyses	Minutes in View per Hour	Naming Instances per Hour
airplane	Not Selected	0.01	0.06
apple	Top Name	0.27	0.84
ball	Not Selected	1.14	0.42
balloon	Not Selected	0.02	0.18
banana	Top Name	0.11	1.08
bed	Not Selected	0.33	0.30
bee	Not Selected	0.08	0.36
bib	Top Name	0.65	1.08
bicycle	Not Selected	0.15	0.36
bird	Not Selected	0.20	0.12
blanket	Top Object	2.02	0.06
block	Not Selected	0.41	0.06
book	Top Name & Object	4.50	2.33
boots	Not Selected	0.18	0.12
bottle	Top Object	11.55	0.48
bowl	Top Name & Object	15.56	1.08
box	Top Object	5.11	0.18
bread	Top Name	0.36	1.20
bug	Not Selected	0.12	0.06
bunny	Not Selected	0.02	0.12
bus	Not Selected	0.01	0.06
butterfly	Not Selected	0.05	0.06
button	Top Name	0.85	0.72
candy	Not Selected	0.07	0.06
car	Not Selected	0.26	0.36
carrots	Top Name	0.16	1.43
cat	Not Selected	0.41	0.42
cereal	Not Selected	0.61	0.42
chair	Top Name & Object	26.12	0.84
cheerios	Not Selected	0.24	0.18
cheese	Top Name	0.42	2.51
chicken	Not Selected	0.01	0.66
clock	Not Selected	0.44	0.06
coat	Not Selected	1.07	0.06
cookie	Not Selected	0.03	0.24
couch	Top Object	4.19	0.30
cracker	Top Name	0.17	0.96
cup	Top Object	13.31	0.66
diaper	Not Selected	0.13	0.42
dog	Top Name	1.19	1.49
door	Top Object	10.15	0.48
drawer	Top Object	3.02	0.24
drink	Not Selected	0.05	0.66
duck	Not Selected	0.09	0.18
egg	Top Name	0.20	3.17
fish	Not Selected	0.02	0.54
fork	Not Selected	1.24	0.66
frog	Not Selected	0.08	0.06
glass	Top Object	7.02	0.18
glasses	Top Object	5.15	0.12
horse	Not Selected	0.01	0.06
house	Top Name	0.22	1.31
icecream	Not Selected	0.06	0.12
jacket	Top Object	2.69	0.18
juice	Top Name	0.18	1.85
kitty	Not Selected	0.01	0.18
lion	Not Selected	0.01	0.06
medicine	Not Selected	0.18	0.12
milk	Top Name	0.42	1.55
moon	Not Selected	0.01	0.48
orange	Not Selected	0.13	0.54
oven	Not Selected	1.98	0.12
pants	Top Object	9.94	0.18
paper	Top Name & Object	6.69	2.45
peas	Not Selected	0.02	0.54
pen	Not Selected	0.20	0.30
pillow	Top Object	2.03	0.12
pizza	Top Name	0.09	1.02
plant	Not Selected	1.45	0.12
plate	Top Name & Object	7.16	1.49
potty	Not Selected	0.02	0.24
shirt	Top Name & Object	27.89	1.20
shoe	Not Selected	2.00	0.54
sink	Top Object	2.15	0.24
sky	Not Selected	0.14	0.06
soap	Not Selected	0.16	0.06
sock	Not Selected	1.25	0.30
spoon	Top Name & Object	7.87	1.55
sun	Not Selected	0.07	0.06
swing	Not Selected	0.03	0.06
table	Top Name & Object	29.60	1.55
telephone	Top Name & Object	2.51	1.02
towel	Top Object	3.60	0.06
toy	Top Name & Object	7.85	1.08
tree	Not Selected	0.78	0.30
truck	Not Selected	0.09	0.06
tv	Not Selected	0.94	0.12
water	Top Name	0.49	2.15
window	Top Object	17.13	0.12
